# Prolonged length of stay in acute psychiatric wards: A descriptive study

**DOI:** 10.1192/j.eurpsy.2021.343

**Published:** 2021-08-13

**Authors:** L. Lopes, M. Gonçalves-Pinho, S. Pereira, J.P. Ribeiro, A. Freitas

**Affiliations:** 1 Department Of Psychiatry And Mental Health, Centro Hospitalar de Vila Nova de Gaia e Espinho, Vila Nova de Gaia, Portugal; 2 Cintesis – Center For Health Technology And Services Research, Faculty of Medicine, University of Porto, Porto, Portugal; 3 Department Of Psychiatry And Mental Health, Centro Hospitalar do Tâmega e Sousa, Penafiel, Portugal, Penafiel, Portugal; 4 Department Of Community, Information And Health Decision Sciences (medcids), Faculty of Medicine, University of Porto, Porto, Portugal, Porto, Portugal

**Keywords:** length of stay, Administrative Database, psychiatric hospitals, Healthcare utilization

## Abstract

**Introduction:**

The psychiatric care paradigm has shifted towards community-centered models. Yet, prolonged hospitalizations are still a reality, with debated impact at healthcare systems and patients.

**Objectives:**

This work aims to describe prolonged hospitalizations in acute psychiatric wards through patients’ sociodemographic and clinical data.

**Methods:**

We analyzed a national hospitalization database that contained all hospitalization episodes registered in Portuguese public hospitals from 2008 to 2015. All episodes with a primary diagnosis of mental disorder defined as ICD-9-CM codes 290.x-319.x were included. Prolonged hospitalizations were defined as having a LoS ≥ P97.5; LOS ≥180 days or LOS ≥1 year. Age, sex, lengh of stay, in-hospital mortality were analysed.

**Results:**

The LoS ≥ P97.5(≥62 days) group comprised 3911 hospitalizations (2.3% of all psychiatric hospitalizations) and 1755 patients. The median LOS was 81 days and the mean age was 51 years. Sex was equally distributed, though a higher frequency of male patients was found on the ≥180 days (n=364) and ≥ 1 year (n=121) groups. Psychotic disorders were the main diagnosis at discharge (n= 1769, 45.2%), followed by mood disorders (n=1057, 27.0%) and dementia (n=451, 11.5%). In-hospital mortality increased in the higher LoS groups (1.1%; 4.4%; 9.1%, respectively).

**Conclusions:**

Overall, middle aged patients with psychotic disorders represent most of the prolonged hospitalizations occurring in acute psychiatric wards. Community-based programs require further development to meet the existing needs.

**Disclosure:**

No significant relationships.

